# Merits and challenges of iPSC-derived organoids for clinical applications

**DOI:** 10.3389/fcell.2023.1188905

**Published:** 2023-05-26

**Authors:** Ziran Xu, Jiaxu Yang, Xianyi Xin, Chengrun Liu, Lisha Li, Xianglin Mei, Meiying Li

**Affiliations:** ^1^ The Key Laboratory of Pathobiology, Ministry of Education, Jilin University, Changchun, Jilin, China; ^2^ Department of Clinical Laboratory, Lequn Branch, The First Hospital of Jilin University, Changchun, Jilin, China; ^3^ Department of Neonatology, The First Hospital of Jilin University, Changchun, Jilin, China; ^4^ Department of Pediatric Cardiovascular Medicine, The First Hospital of Jilin University, Changchun, Jilin, China; ^5^ Department of pathology, The Second Hospital of Jilin University, Changchun, Jilin, China

**Keywords:** IPSC, disease model, 3D, organoid, COVID-19

## Abstract

Induced pluripotent stem cells (iPSCs) have entered an unprecedented state of development since they were first generated. They have played a critical role in disease modeling, drug discovery, and cell replacement therapy, and have contributed to the evolution of disciplines such as cell biology, pathophysiology of diseases, and regenerative medicine. Organoids, the stem cell-derived 3D culture systems that mimic the structure and function of organs *in vitro*, have been widely used in developmental research, disease modeling, and drug screening. Recent advances in combining iPSCs with 3D organoids are facilitating further applications of iPSCs in disease research. Organoids derived from embryonic stem cells, iPSCs, and multi-tissue stem/progenitor cells can replicate the processes of developmental differentiation, homeostatic self-renewal, and regeneration due to tissue damage, offering the potential to unravel the regulatory mechanisms of development and regeneration, and elucidate the pathophysiological processes involved in disease mechanisms. Herein, we have summarized the latest research on the production scheme of organ-specific iPSC-derived organoids, the contribution of these organoids in the treatment of various organ-related diseases, in particular their contribution to COVID-19 treatment, and have discussed the unresolved challenges and shortcomings of these models.

## Graphical Abstract

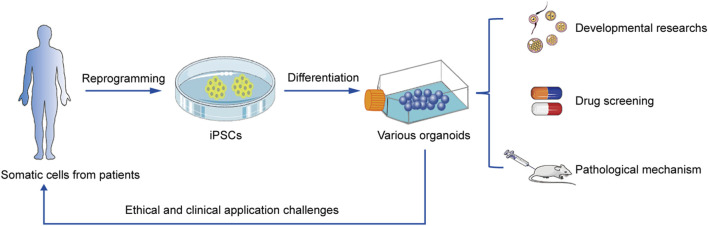

## 1 Introduction

Embryonic stem cells (ESCs) are widely accepted as an important source of cells in the treatment of many degenerative diseases, such as Parkinson’s disease (PD), Alzheimer’s disease (AD), and spinal cord injury, by cell replacement therapy (CRT) (Doss et al., 2004; Ohnuki and Takahashi, 2015; Cyranoski, 2018). However, human ESC research is greatly affected by the use of human embryo-derived cells and the ethical issues arising from the failure of *in vitro* fertilization and destruction of early embryos, as well as the immunologic rejection after transplantation caused by the allogeneic source of ESCs (Tan et al., 2014; Aach et al., 2017). Thus, new experimental techniques are urgently needed to replace human ESCs for experimental research and clinical applications.

James Thompson’s lab created the first human ESC lines and initiated this line of work in 1998 (Thomson et al., 1998). On the basis of this breakthrough experiment, Takahashi and Yamanaka demonstrated how terminally differentiated cells can be reprogrammed to become pluripotent stem cells in 2006 (Takahashi and Yamanaka, 2006). Human fibroblasts could directly enter a pluripotent state by forced ectopic expression of transcription factors (Yu et al., 2007; Aasen et al., 2008; Shi et al., 2008; Haase et al., 2009; Barati et al., 2021). These induced pluripotent stem cells (iPSCs) exhibit similar genetic markers, epigenetic characteristics, and multilineage differentiation potential as ESCs (Shi et al., 2017; Wen et al., 2017). iPSCs can thus be used as a new source of autologous cells for CRT in various degenerative diseases. There has been continuous advances in iPSCs-based disease modeling with extensive research being carried out in this technology. The main advantage of this technique is that iPSCs can be isolated from any individual with a particular disease and large numbers of cells can be derived with clinically relevant phenotypes, without the ethical and immune rejection concerns associated with ESCs. Human-derived iPSCs cell therapy products have started clinical trials and are evaluating their effectiveness and safety (Mandai et al., 2017; Cyranoski, 2018b).

Most of the existing biomedical research models based on iPSCs are cell-line and animal models (Giandomenico et al., 2019). Cell-line models are simple, economic and therefore most commonly used. However, the 2D culture system of single-layer cells lacks cell-cell and cell-extracellular matrix (ECM) interactions (Kanton et al., 2019). In the process of *in vitro* culture, the heterogeneity of cells, and their *in vivo* characteristics are lost, thus they are unable to reproduce the complex 3D environment and the related *in vivo* signaling pathways (Kanton et al., 2019). Animal models can approximate the physiological functions of humans but are often limited by imaging observations, confounding variables, availability constraints, and biological differences between animals and humans. In recent years, the emerging iPSC-derived organoid models have compensated for the above shortcomings (Trujillo et al., 2019).

**FIGURE 1:**
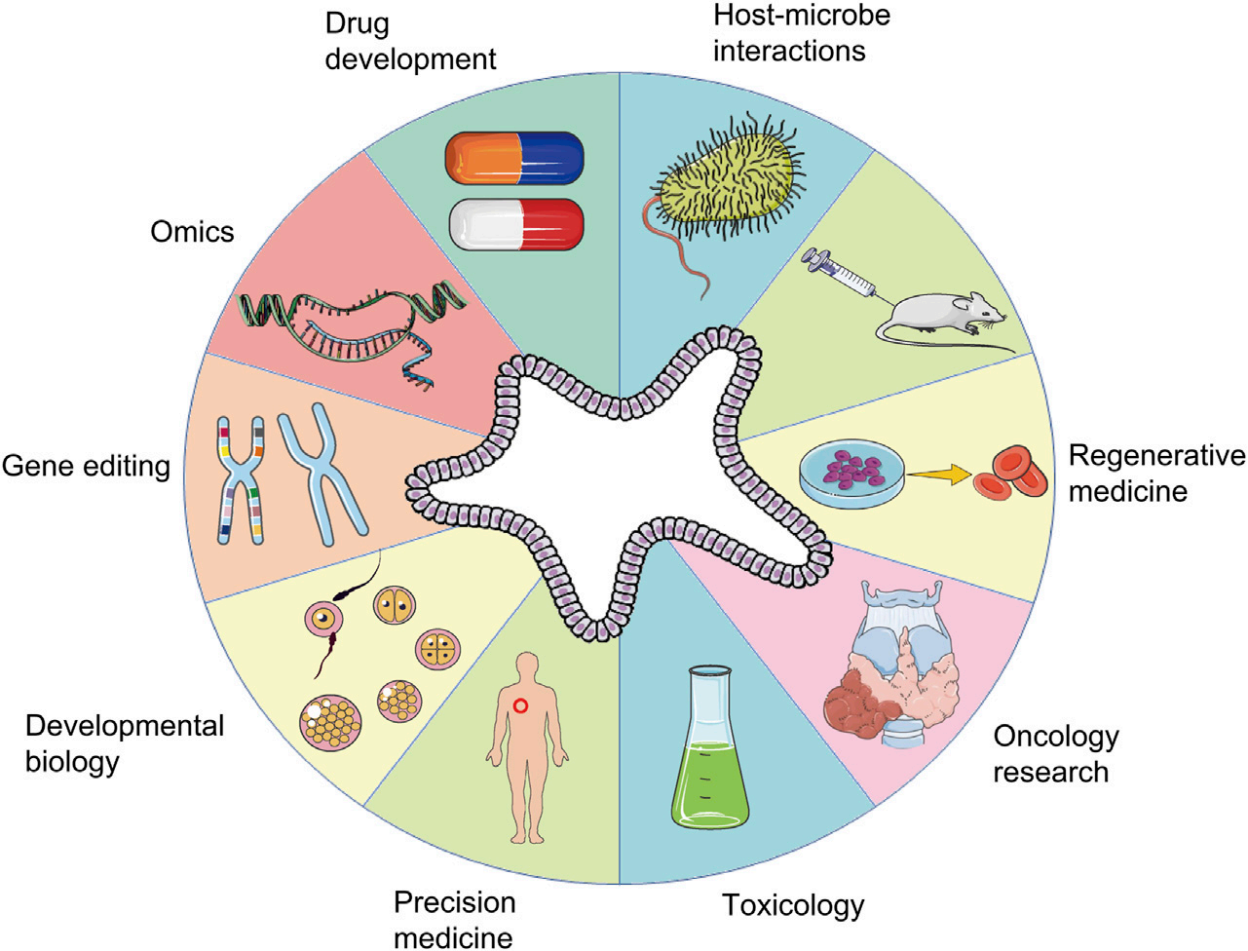
Applications of organoids. A schematic summary of the various applications of organoids, including those in developmental biology, disease modeling, precision medicine, regenerative medicine, toxicology, drug development, oncology research, host-microbe interactions, gene editing, and omics.

The organoid model is an *in vitro* 3D cellular cluster derived exclusively from primary tissue, embryonic stem cells, or induced pluripotent stem cells, capable of self-renewal and self-organization, and exhibiting similar organ functionality as the tissue of origin (Xiang et al., 2019). Different from traditional cell-line models, organoid models can not only be passaged for a long time, but also have stable phenotypic and genetic characteristics (Cakir et al., 2019). For example, liver organoids can stably maintain the phenotypic characteristics of hepatic stem/progenitor cells that can differentiate into functional hepatocytes or cholangiocytes and can be expanded for 20 passages (Wang et al., 2019a). Infectious nasal organoids for studying emerging variants of severe acute respiratory syndrome coronavirus 2 (SARS-CoV-2) can be serially passaged for more than 6 months (Chiu et al., 2022). Cholangiocytes and Small Intestine Organoids can be stably passaged, cryopreserved and recovered (Chen et al., 2022), these advantages of long-term passage preservation make organoids have the basis and advantages to be applied in large-scale clinical research in the future. In the past decade, with advances in *vitro* 3D culture technology, the construction of organoid models has been exploited as an *in vitro* culture system for *in vivo* development procedures, an irreplaceable method to simulate generation of human tissues *in vitro*, which has immense implications in research on disease models, organ regeneration, cell development, and pharmacodynamic testing (Lancaster and Knoblich, 2014; Qian et al., 2020). The application of organoid technology is summarized in Figure 1.

With the development of 3D culture and gene editing technologies, recent advancements in human patient-derived organoids enable precise disease modeling and hold great potential for biomedical applications, translational medicine, and personalized therapy (Dutta et al., 2017), including the coronavirus disease 2019 (COVID-19) disease model, drug screening, and treatment plans. Nevertheless, there are still many challenges that need to be seriously addressed to translate these issues into efficient clinical application in regenerative medicine (Shi et al., 2020). This article mainly introduces disease modeling based on iPSCs and the state-of-the-art progress of iPSCs in organoids modeling, summarizes the challenges faced by the current iPSC technology, and discusses the approaches to solve these problems.

## 2 Disease models based on iPSCs offer new possibilities for *in vitro* modeling of human diseases

It is essential for an experimental model used for investigating the pathophysiology of a disease to reproduce its respective pathophysiological and clinical manifestations. Animal models, in which processes that are considered to be sufficiently similar to those in human disease, have been key participants in basic research and drug development (Shi et al., 2008; Shi et al., 2017). However, the findings obtained using animal models are often not replicated in human drug trials, often resulting in the hospitalization and death of patients due to adverse reactions. Consequently, there is a necessity to develop new experimental methods that would contribute to eliminating the incidence of adverse reactions and mortality associated with the side effects of new drugs (Souied et al., 2017). To address this limitation, appropriate *in vitro* human disease models that can be used to effectively characterize the underlying pathophysiological mechanisms would definitely be a more desirable. In addition, a large number of individuals develop adverse reactions to certain drugs, with many of these confirmed (van den Berg et al., 2019; Bouvy et al., 2015). New experimental techniques for *in vitro* modeling of human disease are needed for new drug discoveries and safe pharmacology to reduce adverse reactions and side effects during clinical drug trials.

Common *in vitro* human disease models are based on primary human cells. The major limitation of this modeling technology is the source of amplifiable cells from patients, which are inadequate, making it difficult to conduct disease modeling experiments on a large scale. Therefore, a new approach for comprehensive *in vitro* human disease modeling is urgently needed in disease and drug discovery research.

Disease models based on human iPSCs are promising potential candidates, and have made significant progress in understanding the etiology and pathophysiology of various diseases like PD, AD, and hereditary heart disease among others (Samuel et al., 2013). As an example, for a long time, the lack of cancer-related disease models posed limits for clinical cancer research. iPSCs generated from human cancer cells have in recent times been used to develop *in vitro* carcinogenesis models (Miyoshi et al., 2010; Stricker et al., 2013; Stricker and Pollard, 2014; Alvisi et al., 2018). Some of these iPSC disease models for cancer-related diseases have emerged as useful potential models for studying the occurrence and development of cancer (Kotini et al., 2015; Lee et al., 2015; Mulero-Navarro et al., 2015; Gingold et al., 2016; Papapetrou, 2016; Crespo et al., 2017; Kotini et al., 2017; Shi et al., 2017; Zhou et al., 2017; Chao and Chern, 2018; Kim and Schaniel, 2018; Zhu et al., 2018). Abnormal cell-level phenotypes have been identified in iPSC-derived cells from patients with various diseases, such as reduced axon number and synaptic protein expression in iPSC-derived neurons from patients diagnosed with schizophrenia (Brennand et al., 2011); iPSCs derived from patients with spinal muscular atrophy could not differentiate into normal motor neurons (Ebert et al., 2009); and cardiomyocytes derived from patients with familial long-QT syndrome had prolonged action potentials similar to those in diseased types (Moretti et al., 2010). These phenotypes appear to be robust, replicable, disease-related, detectable in cell culture, and reflect relevant disease characteristics. Parallel comparisons between *in vitro* disease models based on human iPSCs and *in vivo* rodent models can elucidate the pathophysiological mechanisms of disease in basic medical research, new drug development, and pharmacological safety in unprecedented ways (Gao et al., 2021).

## 3 Organoid models based on iPSCs

Although the traditional initial 2D adherent culture system based on iPSCs has been widely used in multiple fields, it nevertheless has certain shortcomings. For example, in experiments related to the brain research, we can neither simulate the microenvironment of the brain, nor simulate the interaction between cells in 3D space (Meyers et al., 2006; Aloysious and Nair, 2014). As a result, the neurological disorders affecting different types of cells in different regions of the brain have not been well-modeled (Mseka et al., 2007). In the research related to the liver, there are complications, such as difficulty in obtaining primary hepatocytes, poor proliferation ability *in vitro*, and cell polarity cannot be maintained for a long time, and only a single type of hepatocyte is involved, while ignoring the important functions of other non-parenchymal cells in the liver. In heart-related studies, cardiac lineage cells cultured in a traditional 2D culture system are quite different from natural cardiomyocytes in terms of morphology, viability, cell function, and gene expression, and it is difficult to reflect the spatial interaction between different cell types and the communication between cells and matrix (Wang et al., 2017). On the basis of the aforementioned limitations and a number of other technical reasons, the emergence of iPSC-derived organoids based on 3D culture systems has been a particularly welcome development.

Organoids are 3D multicellular aggregates derived from stem cells, which can replicate the structural characteristics of mature tissue, and interaction between cells (Ho et al., 2018). Biophysical cues provided to induce iPSC differentiation into ‘tissue in a dish’ are well established (McCauley and Wells, 2017). The basic principle is to supplement a variety of inducing factors at appropriate time and dose, to make the cells with the potential for self-renewal and differentiation from the original cells spontaneously differentiate into part of the corresponding organs, and then use an artificial matrix instead of extracellular matrix, to realize the construction of 3D culture system (Hogberg et al., 2013; Lancaster et al., 2013). The 3D culture system has an extracellular matrix more similar to the natural state than the 2D culture system, and hence can simulate the endogenous microenvironment composed of various autocrine signals, and more accurately simulate the cell morphology, proliferation, migration, differentiation, and other developmental processes (Mimeault et al., 2007).

**FIGURE 2:**
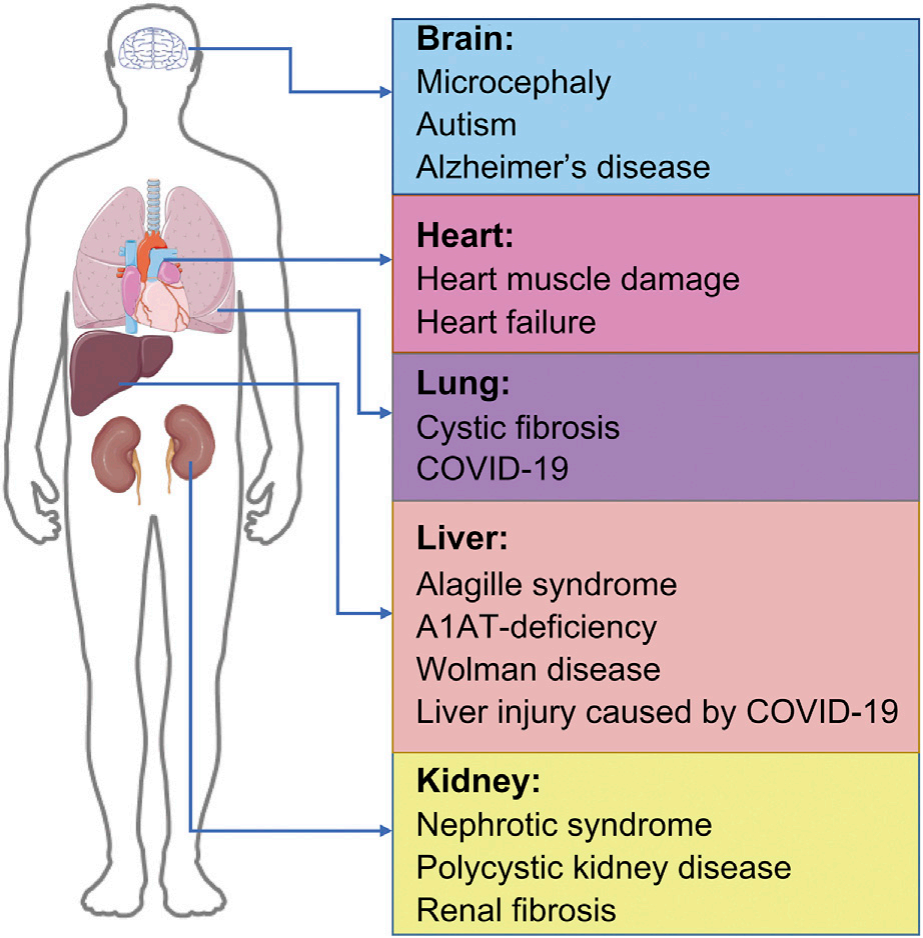
Applications of organoids-based therapy. IPSC-derived organoids and their applications in disease, including brain organoids, cardiac organoids, lung organoids, liver organoids, and kidney organoids. IPSC, induced pluripotent stem cells; COVID-19, coronavirus disease 2019.

In recent times, the increasing longevity and improved health of older adults has contribute to an increasingly aging society, accompanied by an increase in the prevalence of age-related diseases and disability, particularly those involving the brain, liver, heart, kidneys, and lungs, and iPSC organoid technology has been shown to have numerous applications in the treatment of such diseases. COVID-19, an extremely contagious disease caused by the highly pathogenic SARS-CoV-2, has resulted in widespread health problems globally (Huang et al., 2020; Zhu et al., 2020). No specific treatment is yet available for COVID-19, which has an overall fatality rate of approximately 1% with 3%–20% of COVID-19 patients requiring hospitalization (Petersen et al., 2020; Mahajan et al., 2021); this highlights our limited understanding of the disease pathogenesis. Although SARS-CoV-2 primarily targets human lungs (Lamers and Haagmans, 2022), COVID-19 can also cause damage to other organs, including brain, heart, kidney, and liver (Deguchi et al., 2021; Luo et al., 2021), causing complications like mental illness, myocarditis, hypertension, heart failure, cardiac arrhythmia, cardiac arrest, liver injury, diabetes, renal failure, gastrointestinal complications, and chronic lung disease among others; it can also cause arrhythmia, rhabdomyolysis, coagulation disorders and shock (Berlin et al., 2020; Karagiannidis et al., 2020; Han et al., 2022). Since iPSC-derived organoids have made great progress in disease modeling with continuous development (Liu et al., 2018), organoids, which can self-renew and recapitulate various physiologies of different organs, can be a powerful platform for modeling COVID-19. Different iPSC-derived organoids can be produced, including the brain, lung, cardiac, liver, and kidney organoids, respectively. Their contributions to the study and treatment of various diseases have been significant (Figure 2).

### 3.1 Brain organoids

**FIGURE 3:**
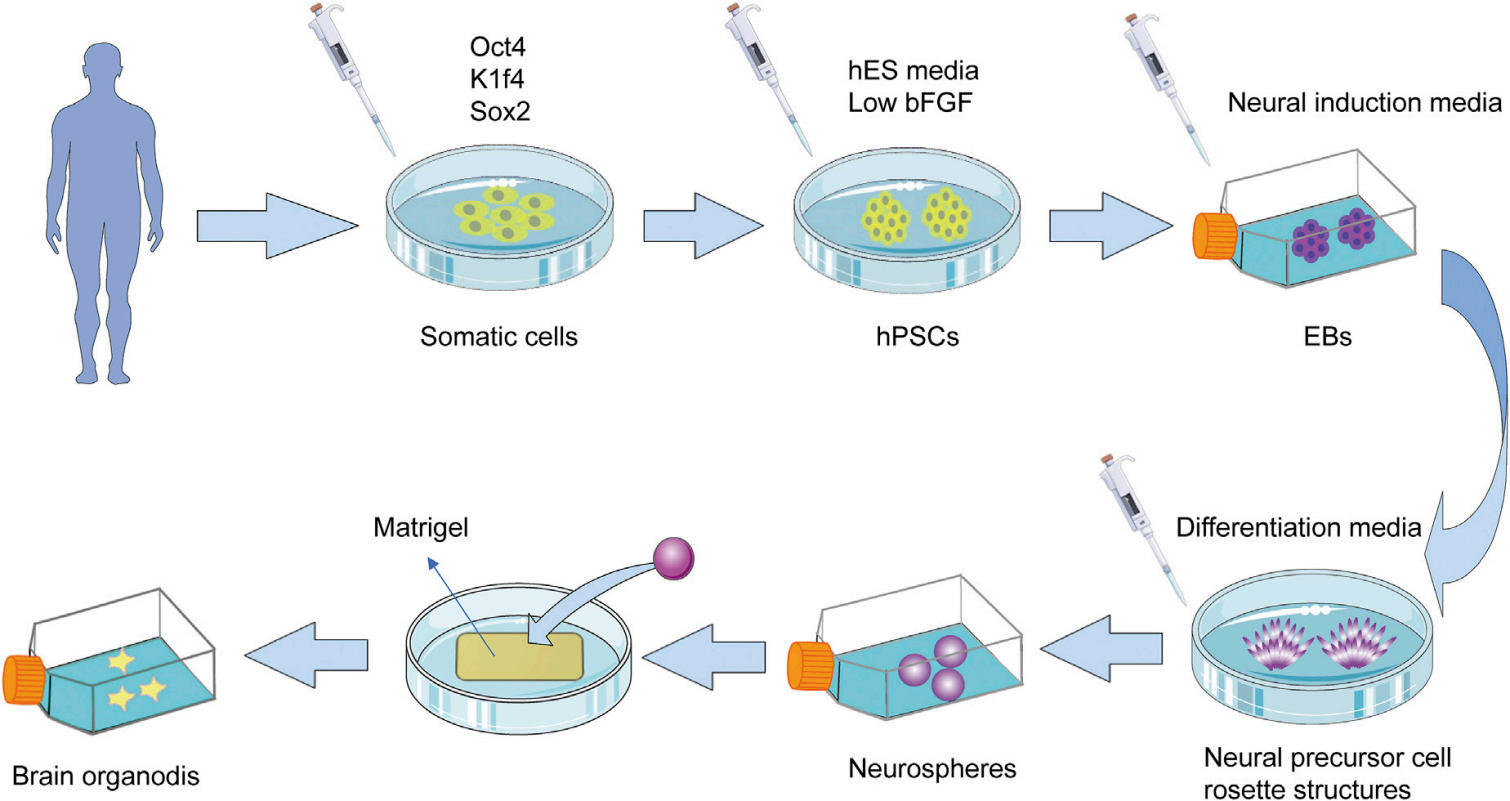
The most widely used scheme for the production of 3D brain organoids. Reprogramming of somatic cells into hPSCs by introducing specific transcription factors such as Oct4, Kfl4, and Sox2. Place the obtained hPSCs into the suspension containing hES media and low levels of bFGF to induce them to form EBs. These suspension-cultured EBs then gradually differentiate into several polarized neural progenitor cell rosette structures, which are re-blown and suspended into neurospheres. The obtained neurospheres are embedded in the Matrigel to produce brain organoids with specific cortical characteristics. These organoids take 8–10 days to develop neural characteristics and 20–30 days to form definite brain regions. hPSC, human pluripotent stem cells; hES: human embryonic stem cell; EB, embryoid body; FGF, fibroblast growth factor.

In brain and neuroscience research, although investigators have developed a 2D nerve culture system that can establish connections between two different brain regions, namely, neocortex, and midbrain neurons, only with the advent of the 3D culture system, it was possible to simulate the formation of synaptic connections (Comella-Bolla et al., 2020). The most widely used scheme for human pluripotent stem cell (hPSCs)-derived 3D brain organoids is to use matrix glue to coat embryoid body (EB)⁃like rapid aggregation (Watanabe et al., 2005; Barzegari et al., 2020), that is, through the formation of EB, these EB-like aggregates in suspension culture gradually differentiate into several polarized neural precursor cell rosette structures, and then re-blow into neurospheres. The brain organoids with specific cortical characteristics are then embedded within matrix glue containing high levels of neuronal extracellular matrix proteins (Figure 3).

In recent years, researchers have constructed brain organoids that replicate parts of the brain tissue or structure, and have attempted to fuse them with other types of organoids. Way back in 2013, Kadoshima’s team developed a polarized cortical self-organizing structure with specific progenitor cell dynamics derived from human ESC, which is close to the present concept of brain-organoid (Kadoshima et al., 2013). It has been shown that transplantation of human brain organoids into adult mouse brains can result in establishment of functional synaptic connections between the two (Mansour et al., 2018). Embedment of human ESCs and human iPSCs in the 3D culture system has been shown to result in cultivation of “whole-brain organoids” similar to the brains of 9–10-week-old embryos, with characteristic structures such as the appearance of dorsal cortex and ventral forebrain (Lancaster et al., 2013). Kirwan et al. (Kirwan et al., 2015) also successfully constructed an organoid model that mimics the development and function of cortical networks using human iPSC lines. Co-cultivation of human iPSC-derived brain organoids with endothelial cells in the Matrigel yielded vascularized brain organoids (Pham et al., 2018). Andersen’s team fused organoids resembling the cerebral cortex and hindbrain/spinal cord, both of which were derived from iPSCs, with skeletal muscle organoids to produce fusion organoids or “assembloids” containing an assembly of cortical descending neurons, spinal cord-derived motor neurons, and muscles (Andersen et al., 2020). These organs can maintain functional contraction *in vitro* for a long time and can be controlled by optogenetic and pharmacological manipulation. Brain organoids have been gradually developed from brain tissue cells to combinations with other cells or organoids to explore more potential applications.

Researchers can now utilize brain organoids to create models of brain development and diseases of nervous system as well as to investigate the causes and progression of neurologicaldisorders, thanks to the development of organoid-3D culture technology. When neural organoids from patient-derived iPSCs were used to emulate microcephaly, for instance, it was discovered that there was premature neuroepithelial differentiation, aberrant radial glial cell orientation, and reduced regions of differentiated neural tissue (Brüstle, 2013; Lancaster et al., 2013). Similar signs were observed in iPSCs of patients with microcephaly caused by centromere protein J mutations, which is another centrosomal protein that plays a regulatory role in microtubule assembly and nucleation (Gabriel et al., 2016). In autistic patients, although iPSCs-derived neural organoids have been established to undergo normal early neuronal differentiation, owing to an overexpression of the transcription factor FOXG1, there is an excessive production GABAergic inhibitory neurons with no change in glutamatergic neurons, thereby indicating that this imbalance in glutamate/GABA neuron differentiation ratio is one of the mechanisms associated with autism (Mariani et al., 2015). IPSC-derived nerve organoids from Rett syndrome patients with mutations in the methyl-CpG-binding protein 2 revealed nerve injury and decreased neuronal migration (Mellios et al., 2018). Apolipoprotein E (APOE) is a kind of lipid transporter, which is widely distributed in the periphery and brain (Serrano-Pozo et al., 2021). The phosphorylation of tau protein in brain organoids carrying *APOE ε4* allele has been shown to be elevated irrespective of whether the organoids are derived from healthy patients or patients with AD. In this regard, the gene editing technique has been used to convert *APOE ε4* to *APOE ε3,* thereby attenuating the *APOE ε4*-related AD pathological phenotype, and thus indicating that *APOE ε4* may serves as a target in the therapy of AD (Zhao et al., 2020a). The application of brain organoids to elucidate the causes and progression of neurological diseases is of great significance for disease treatment and drug development.

At present, much progress has been made in the research on the technology of 3D brain organoids generated from iPSCs. In addition to construction of models for brain development and nervous system disease, this technology is also being used to study the cell division in human radial glial cells and the expansion of human cortical progenitor cells (Otani et al., 2016; Pollen et al., 2019), together with use for drug screening, for example, for prenatal drug treatment (Lee et al., 2011). Scientists have studied the effects of prenatal drug exposure on the human fetal brain using the human brain organoids model and discovered that cocaine exposure might impede the multiplication of neocortical progenitor cells. Cocaine induces early neuronal differentiation and disrupts nerve tissue development, and it has been discovered that cytochrome P4503A5 may be a therapeutic target for the treatment of neurodevelopmental problems caused by prenatal cocaine exposure (Lee et al., 2017). This shows that brain organoids can help in searching for appropriate drug treatment targets and provide a new platform for perinatal drug research.

The COVID-19 pandemic’s spread has slowed, but is not yet over. Clinical observations have frequently detected neurological signs and neuropsychiatric disorders related to the disease (Bodnar et al., 2021). The latest research reported that attacking iPSCs-induced human brain organoids with SARS-CoV-2 spike pseudo-virus and the live virus can directly target cortical neurons and neural progenitor cells (Zhang et al., 2020), infect the choroid plexus and destroy the blood-cerebrospinal fluid barrier, thereby explaining the causes of headache, epilepsy, confusion and other neurological symptoms in clinical patients in addition to respiratory symptoms (Jacob et al., 2020; Pellegrini et al., 2020; Varatharaj et al., 2020). Yale University researchers used brain organoids to discover that SARS-CoV-2 can directly invade the central nervous system (CNS), infect neurons via the angiotensin-converting enzyme 2 (ACE2) receptors, and promote the replication of the infection-related metabolism in the invaded neurons, causing adjacent uninfected neurons to die due to hypoxia. Also, SARS-CoV-2 infection of the CNS was more deadly than lung infection in mice (Song et al., 2020). Other studies had shown that in human brain organoids infected with SARS-CoV-2, the virus could promote neuronal cell death, including cortical neurons, and the antiviral drug Sofosbuvir could inhibit SARS-CoV-2 replication, and rescue of these neuronal alterations in infected brain organoids (Mesci et al., 2022). These studies aid to guide a rational approach and screening for antiviral drugs to treat patients with COVID-19 with neurological symptoms.

Despite these advancements in the production of 3D brain organoids from iPSCs, there are still certain issues to be resolved. Brain organoids are not real human brains and cannot self-organize themselves into exactly the same shape and functional zones as the human brain. At the same time, the development and maturity of the brain, especially in the late stage, are highly dependent on the vascularization of the subventricular zone. In absence of formation of blood vessels, the organ’s supply of oxygen and nutrients becomes limited, which often causes necrosis in the central area and interferes with the migration of neurons (Nam et al., 2020). Researchers found that compared with other brain tissues, though cortical organoids contain a wide range of cell types, their radial glial cells and intermediate progenitor cells are reduced, as is the content of cell subtypes such as upper neurons, cell subtypes are damaged, cell stress pathways are abnormal, and fidelity is disturbed (Bhaduri et al., 2020). Therefore, although many cell types have been differentiated from brain organoids, there are still differences between them and the primary cells. But this will not affect the significance of 3D brain organoid technology research on the brain development process, occurrence and development of nervous system diseases, nerve cell division, drug screening, and other aspects.

### 3.2 Liver organoids

To create liver organoids, Takebe et al. (Takebe et al., 2013) reproduced the formation of 3D vascular and functional iPSC-derived liver buds (iPSC-LBs) *in vitro* for the first time. They first co-cultured the human-iPSC-derived hepatic endodermal cells with stromal cells, and then transplanted them into immunodeficient mice after forming liver buds. The blood vessels in iPSC-LBs could connect with the host blood vessels, the LBs induced *in vitro* were similar to those *in vivo*, and their functional blood vessels could stimulate the development of iPSC-LBs into mature liver tissue. Subsequently, this research group established a large-scale organoid culture platform, which can efficiently produce homogeneous small LBs (Takebe et al., 2017). After that, the same research group made another breakthrough when they used iPSCs to create continuous and dynamic liver-gallbladder-pancreatic organoids (Koike et al., 2019). Briefly, they established a region-specific intestinal “spheroid” structure of endoderm and mesoderm by the 3D culture of human pluripotent stem cell, and then embedded two “globules” adjacent to the matrix gel to promote spheroid fusion, and then liver-gallbladder-pancreatic primordia appeared at the fusion interface. After long-term culture, organ primordia can reproduce morphogenetic events in the early stage of development, including invagination, branching, and connection, resulting in the formation of mini liver-gallbladder-pancreas organoids. The functional connection is established between the internal pancreas (especially the exocrine pedigree) and the bile duct (Koike et al., 2019) This study provides the possibility for the study of complex interactions in the early process of organ development. In 2020, the University of Pittsburgh team differentiated human iPSCs into different liver cell lines, resulting in a fully functional artificial miniature liver that could survive for 4 days in rats (Takeishi et al., 2020). This study is a remarkable progress in the field of artificial organ replacement in laboratory culture. In the same year, Velazquez et al. (Velazquez et al., 2021) constructed human liver organoids with vascularization and four main cell types (including hepatocytes, bile duct cells, endothelial cells, and stellate cells) *in vitro* through gene regulatory network technology and Clustered Regularly Interspaced Short Palindromic Repeats technology, and these organoids were similar to adult human liver function in energy storage, chemical transport, fat accumulation, enzyme activity, and protein production (Velazquez et al., 2021).

Utilizing 3D organoid systems, bile duct cells produced from iPSCs derive from patients with hepatobiliary disorders have been developed (Ogawa et al., 2015; Sampaziotis et al., 2015). Two differentiation protocols that develop from the beginning to the final endoderm and hepatocyte, and subsequently to bile duct-like cells, have been reported. (Ogawa et al., 2015; Sampaziotis et al., 2015). These bile duct organelles excrete bile acids and secrete bile acids functionally, making it possible to establish a model of Alagille syndrome. The synthetic somatostatin analog octreotide lowers the size of bile duct organoids differentiated from individuals with polycystic liver disease, which is consistent with the impact of octreotide in patients with polycystic liver disease. Bile duct organelles isolated from iPSCs from cystic fibrosis (CF) patients with a phenylalanine deletion at position 508 (F508del) of the CF transmembrane conductance regulator (CFTR) showed impaired chlorine transport and reduced swelling induced by CFTR-dependent forskolin, which was rescued by CFTR correcting drug VX-809 (Ogawa et al., 2015; Sampaziotis et al., 2015). Autosomal recessive Wolman disease is caused by the inactivation of acid lipase in lysosomes. There is a large accumulation of lipids in the liver cells of the patients, accompanied by fatal steatohepatitis and fibrosis. To explore a new treatment, the Ouchi et al.‘s team at Tokyo Medical University used Wolman disease patient-specific iPSC line-derived organoids to construct 3 organoid models with severe fibrosis. Obeticholic acid (OCA) can relieve Wolman’s symptoms by stimulating the production of fibroblast growth factor (FGF) 19 in the ileum. After adding FGF19 to the organ culture-like system for 7 days, the level of reactive oxygen species, a hallmark of hepatocyte damage in non-alcoholic fatty liver, was dramatically reduced, as was the degree of organoid fibrosis. This study confirmed the potential value of FGF19 and OCA in the treatment of Wolman disease and provides ideas for the development of new drugs (Ouchi et al., 2019).

Liver injury is also a coexisting symptom reported in patients with COVID-19. An epidemiological investigation found that 55 (37.2%) of the 148 patients with COVID-19 had abnormal liver function, and the key liver function indices, such as alanine aminotransferase, aspartate aminotransferase, alkaline phosphatase, or total bilirubin, were higher than normal (Fan et al., 2020). Organoids derived from various tissue sources can be utilized to detect locations susceptible to SARS-CoV-2 infection and can aid in research, diagnosis, and therapy planning. It has been reported that SARS-CoV-2 may infiltrate liver ductal organs, reproduce and spread in enormous numbers (Zhao et al., 2020b). The findings also reveals that liver injury in individuals with COVID-19 may be caused by damage to bile duct cells induced by viral infection and subsequent bile acid buildup. The fundamental mechanism is that SARS-CoV-2 infection reduces Claudin1 gene expression and damages bile duct cell barrier function. Most critically, following SARS-CoV-2 infection, the expression of two key bile acid transporters, apical sodium-dependent bile acid transporter and CFTR protein, was considerably reduced, resulting in a reduction in bile acid transport function (Zhao et al., 2020b). Therefore, the treatment of COVID-19 liver injury could be based on the pathogenic mechanism obtained from the study of liver organoids, such as targeting the associated gene or related protein content for corresponding treatment and prevention.

### 3.3 Kidney organoids

Nishinakamura’s group (Taguchi et al., 2014) successfully induced iPSCs into kidney organoids. The EB was induced by bone morphogenetic protein 4 (BMP4) treatment, and subsequently the posterior mesoderm was formed under the combined induction with activin, BMP4, Wnt pathway agonist (CHIR99021), retinoic acid, and FGF 2. After adding CHIR99021 and FGF 9, the postrenal interstitium was obtained. Co-culturing with mouse spinal cord, proximal and distal tubules and podocyte-like structures could be observed in the organoids. Around the same time, hESCs and hPCs could be induced into kidney organoids with nephrons and ureteric bud structures, respectively (Xia et al., 2013; Takasato et al., 2014). In 2015, Freedman et al. established a special renal organ differentiation program. In this scheme, only CHIR99021 induction was added for 36 h and the “sandwich” culture method was used. The stem cells cultured in this way spontaneously aggregate to form hollow spheres. On the 21st day or so, tubular renal organoids containing proximal renal tubules, distal renal tubules, and podocytes are formed (Freedman et al., 2015). This scheme was later improved into a fully automated and high-throughput method for producing of kidney organoids (Czerniecki et al., 2018). This approach offers the advantages of simple steps and low cost when compared to other schemes, however there are more off-target differentiation cells due to the use of only one induction reagent. Low et al. (2019) found a scheme for the differentiation of vascularized kidney organoids, which is caused by precise control of the ratio of glomeruli to tubules. Kidney organoids are being continuously optimized in protocols forimprovements.

Kidney organoids have extensive applications in disease modeling. Congenital nephrotic syndrome is a severe hereditary nephropathy. Nishinakamura’s team (Tan et al., 2014) reprogrammed the patient’s skin fibroblasts to obtain iPSC and induced the formation of kidney organoids, and found that in the podocytes of these organoids, the protein nephrin encoded by *nephrosis 1* gene could not be located on the cell surface, thus affecting the formation of pore membrane. After correcting the missense mutation carried by the patient and re-inducing to form an organoid, the cell surface localization ability and phosphorylation state of nephrin were restored, and the two proteins, IRRE-like protein 1 and podocin could be recruited normally, thus forming a complete hiatal membrane structure. Kidney organoids can also simulate the process of renal fibrosis *in vitro*, track cell fate, and understand the mechanism of renal injury, which is helpful for the study of renal fibrosis and the treatment of chronic kidney disease (Hale et al., 2018).

Drug-induced nephrotoxicity is an inevitable adverse drug reaction in hospital settings. The expression of certain nephrotoxic drug target proteins in renal tubular epithelial cells is critical in the reabsorption of several nephrotoxic medicines, such as cisplatin and gentamicin. Scientists detected the nephrotoxicity of these two drugs using the proximal and distal tubules of kidney organoids induced by iPSCs *in vitro*. After treatment with gentamicin 5 mg/mL and cisplatin of 5 mmol/L, the expression of renal injury molecule 1 was highly upregulated in proximal and distal tubules (Morizane et al., 2015). In addition, researchers tested the nephrotoxicity of tacrolimus *in vitro* using kidney organoids derived from human iPSCs. The proximal tubular cells of kidney organoids treated with tacrolimus contained a large number of vacuoles and showed characteristics of oxidative stress, mitochondrial dysfunction and increased autophagy activity (Kim et al., 2021). Thus, kidney organoids can provide research models closer to human physiology for testing drug nephrotoxicity.

Kidney failure is often observed during, and after COVID-19. In a recent study, researchers infected iPSC-derived kidney organoids with SARS-CoV-2, and found that similar to tissues in patients with COVID-19, kidney organoids developed scars, which were accompanied by signals that led to scar formation. Single-cell RNA sequencing showed that the damage and dedifferentiation of infected cells were related to the activation of the fibrotic signal pathway. Significantly, SARS-CoV-2 infection causes an increase in collagen 1 expression in organoids. A SARS-CoV-2 protease inhibitor can also ameliorate SARS-CoV-2 infection in kidney cells. These findings reveal that SARS-CoV-2 may directly infect renal cells, causing cell damage and eventual fibrosis with no involvement of the immune system (Jansen et al., 2022). This finding has important implications for the pathogenesis of kidney failure caused by COVID-19 and can guide future treatment strategies.

### 3.4 Cardiac organoids

Cardiac development can be divided into two stages: cardio genesis and maturation. In the early stage of cardio genesis, embryonic cardiogenic mesoderm cells undergo typing and differentiation to form a complex cardiac structure, while in the late mature stage, the heart will undergo some adaptive changes to meet the functional needs (Klaus et al., 2007; Wang et al., 2019b; Guo and Pu, 2020). iPSCs mainly simulate these two processes *in vitro*. Earlier, scientists used the strategy of EB differentiation to construct cardiac organoids (Lancaster and Knoblich, 2014). In the early stage of development, the embryonic heart is regulated by a variety of signal pathways such as Wnt, BMP, and Notch from surrounding tissues. Wnt signal is not only a key signal pathway involved in the development of cardiac mesoderm, but also an important regulatory pathway for PSC-derived cardiac lineage cells and the construction of cardiac organoids (Trillhaase et al., 2021). Therefore, when constructing cardiac organoids *in vitro*, it is necessary to add additional exogenous cardiogenic factors to simulate the environment for heart development *in vivo* (Andersen et al., 2018). Researchers from Hanover Medical College in Germany embedded iPSC aggregates into matrix glue and treated them with Wnt signal pathway activator CHIR and inhibitor IWP2 successively, and successfully obtained cardiac organoids which are very similar to the early heart structure, which can be used to study the early development of foregut and heart (Drakhlis et al., 2021). In the same year, Rossi et al. created embryoids with heart-like tissue and formed beating heart tissue (Rossi et al., 2021), Later, Lewis-Israeli et al. and Hofbauer et al. described the most advanced cardiac organoids to date, they contain more complex lumen structures, both of which are useful in the study of congenital heart defects (Hofbauer et al., 2021; Lewis-Israeli et al., 2021). The latest reports represent a major effort to improve and mature heart organoids, a step forward in creating a fully synthetic human heart (Volmert et al., 2022). In addition to the EB differentiation strategy, scientists have also established other methods for the construction of human cardiac organoids, which mainly involve cell re-agglutination and tissue micropatterning (Yin et al., 2016). Agglutination is usually formed by the self-organization of cardiac lineage cells derived from iPSCs in biomaterials with 3D structure (Giacomelli et al., 2020). The micro-pattern method mainly depends on lithography technology, using polydimethylsiloxane combined with other biomaterials to make controllable microstructure molds (Zhao et al., 2019). This kind of micro-mold can be prepared into different sizes and shapes according to the needs of the experiment, so that the differentiated heart pedigree cells can form a specific tissue morphology after inoculation. Scientists have successfully constructed different models of human heart disease from iPSCs, which has been helpful for studying cardiovascular diseases.

In a study, researchers injected iPSC-derived cardiomyocytes wrapped with collagen gel into a circular mold to form human cardiac organoids, which were then locally frozen to simulate acute myocardial injury *in vivo*. The study found that the function of human cardiac organoids recovered 2 weeks after acute injury (Voges et al., 2017). This was the first study to show that human cardiac organoids resemble fetal/neonatal cardiac tissues and can exhibit an endogenous regeneration response, which provided a good perspective for the study of heart regeneration and its molecular mechanism. Tiburcy et al. (Tiburcy et al., 2017) successfully constructed human cardiac organoids with more mature cardiomyocyte structure and function under serum-free conditions. Using this model, they found that overstimulation of neurohumoral fluids (such as catecholamines) can result in contractile dysfunction of human cardiac organoids, myocardial hypertrophy, cardiomyocyte death, adrenergic signal desensitization, and the release of heart failure biomarkers. These characteristics are typical manifestations of heart failure. β-adrenoceptor antagonist metoprolol and α-adrenoceptor antagonist phenoxybenzamine could partially inhibit the pathological phenotype of human cardiac organoids induced by catecholamine. This study laid a theoretical foundation for the application of human cardiac organoids in the study of heart failure models and evaluation of anti-heart failure drugs, and is likely to be helpful in developing new treatment strategies for heart failure.

It was shown that there is significant immunosuppression in patients with severe early COVID-19, which may lead to cytokine storm (CS) and organ damage in the later stages of the disease (Tian et al., 2020). Inflammatory mediators such as tumor necrosis factor can cause systolic dysfunction and therefore, high inflammation may play an important role in heart damage and dysfunction (Feldman et al., 2000). In a recent study (Mills et al., 2021), all paired combinations of inflammatory factors were screened in cardiac organoids derived from hPSCs through a variety of functional metrics. It was found that the combined induction of interferon- γ, interleukin-1 β, and polyiridol C can cause severe diastolic dysfunction. The researchers continued to measure cardiac organoid samples using phosphorylated proteomics techniques, focusing on diastolic dysfunction caused by CS. The results showed that there was a strong viral stress response in many cell populations of the heart, which is speculated to be mediated by epigenetic activation of signal transducer and activator of transcription 1 and bromodomain-containing protein (BRD)4. The researchers then evaluated three bromodomain and extra-terminal family inhibitors (BETi) in the Food and Drug Administration (FDA) compound library and found that BETi prevented CS-induced diastolic dysfunction in a dose-dependent manner, protected dysfunctional cardiac organoids and restored diastolic function 24 h after CS episode. Thus, it was deduced that CS-induced diastolic dysfunction is reversible and is caused by the presence of inflammatory substances. In conclusion, it is proposed that CS promotes diastolic dysfunction via a BRD4-dependent mechanism that may be inhibited by BETi. BETi can reduce the expression of ACE2 and reduce SARS-CoV2 infection. This study can be of guiding significance for acquiring knowledge about the mechanism and subsequent treatment of cardiac injury caused by COVID-19.

### 3.5 Lung organoids

To construct lung organoids, iPSCs were first differentiated into the stereotyped endoderm stage under the action of activin A, and then cytokines and small molecular compounds were added to further differentiate the cells in the stereotyped endoderm stage into the anterior intestinal endoderm stage containing pulmonary progenitor cells. These developmental progenitor cells then formed the foregut spheroids, and finally differentiated into lung-like organoids in 3D culture environment (Huang et al., 2014). This kind of organoid contains many cell types, such as lung alveolar type 2 (AT2) cells, type I alveolar epithelial cells, stromal cells, proliferating cells and so on. More importantly, AT2 cells in this pulmonary organoid express ACE2 and transmembrane serine protease 2, which is consistent with human lung RNA-sequencing results.

Recent studies have emphasized the role of human iPSCs-derived lung organoids in disease modeling by directionally differentiating human iPSCs into airway progenitor cells that are capable of subsequently forming either proximal or distal airway cells (McCauley et al., 2017; Chang et al., 2021). In 3D culture at low levels of Wnt activators, airway progenitor cells can repeatedly form organoids with cells containing various proximal airway cells, including secretory, goblet and basal cells (Aisenbrey et al., 2021). IPSC-derived proximal airway-like organoids from homozygous patients with CF F508del-CFTR mutations and control healthy humans were treated with forskolin, and it was observed that the patient-derived organoids showed impaired forskolin-induced swelling (McCauley et al., 2017). This phenotype was attributed to CFTR malfunction, and the subsequent gene editing of F508del in the iPSCs improved forskolin-induced swelling (Alsayegh et al., 2019), confirming the genotypic-phenotypic relationship in iPSCs disease models (Ashmore-Harris et al., 2019). In 3-D culture, human iPSC-derived lung progenitor cells were able to develop into distal alveolar organoids including functioning type 2 alveolar epithelial cells with lamellar bodies, secreting surface-active proteins (Chen, 2020). For iPSCs from patients lacking surfactant protein B (SFTPB), although the derived alveolar organoids contain type 2 alveolar epithelial cells, these cells lack lamellar bodies or cannot synthesize SFTPB (De Los Angeles and Tunbridge, 2020). The results of the above studies suggest that correction of SFTPB mutations by gene editing may alter the phenotype of specific patient-derived iPSCs (Bogacheva et al., 2021). These studies suggest that human iPSC-derived lung organoids have potential applications in modeling and drug screening for lung diseases.

Human lung organoids are a valuable preclinical model for investigating COVID-19 pathophysiology and treatment development. Many organizations have established organoid cultures to explore the SARS-CoV-2 infection response. Youk et al. (2020) created a 3D model in which human lung AT2 cells were grown into organoids and then cut into pieces to enhance viral access to the cells’ apical surface. They discovered that SARS-CoV-2 successfully infected this hAT2 cells, resulting in an innate immune response. Hou et al. discovered that infection caused cell-autonomous and non-cell-autonomous apoptosis in alveolar spheres of distal lung epithelial cells mixed with human embryonic lung fibroblasts, which may contribute to alveolar damage. They also examined infection of proximal airway epithelial cells in air-liquid interface cultures and discovered that the virus predominantly targeted ciliated airway epithelial cells, which was similar with findings in COVID-19 *postmortem* lungs (Hou et al., 2020). This showed that human lung organoids could be used as an *in vitro* model to observe the occurrence and development of lung injury disease after infection with the SARS-CoV-2 virus.

**FIGURE 4:**
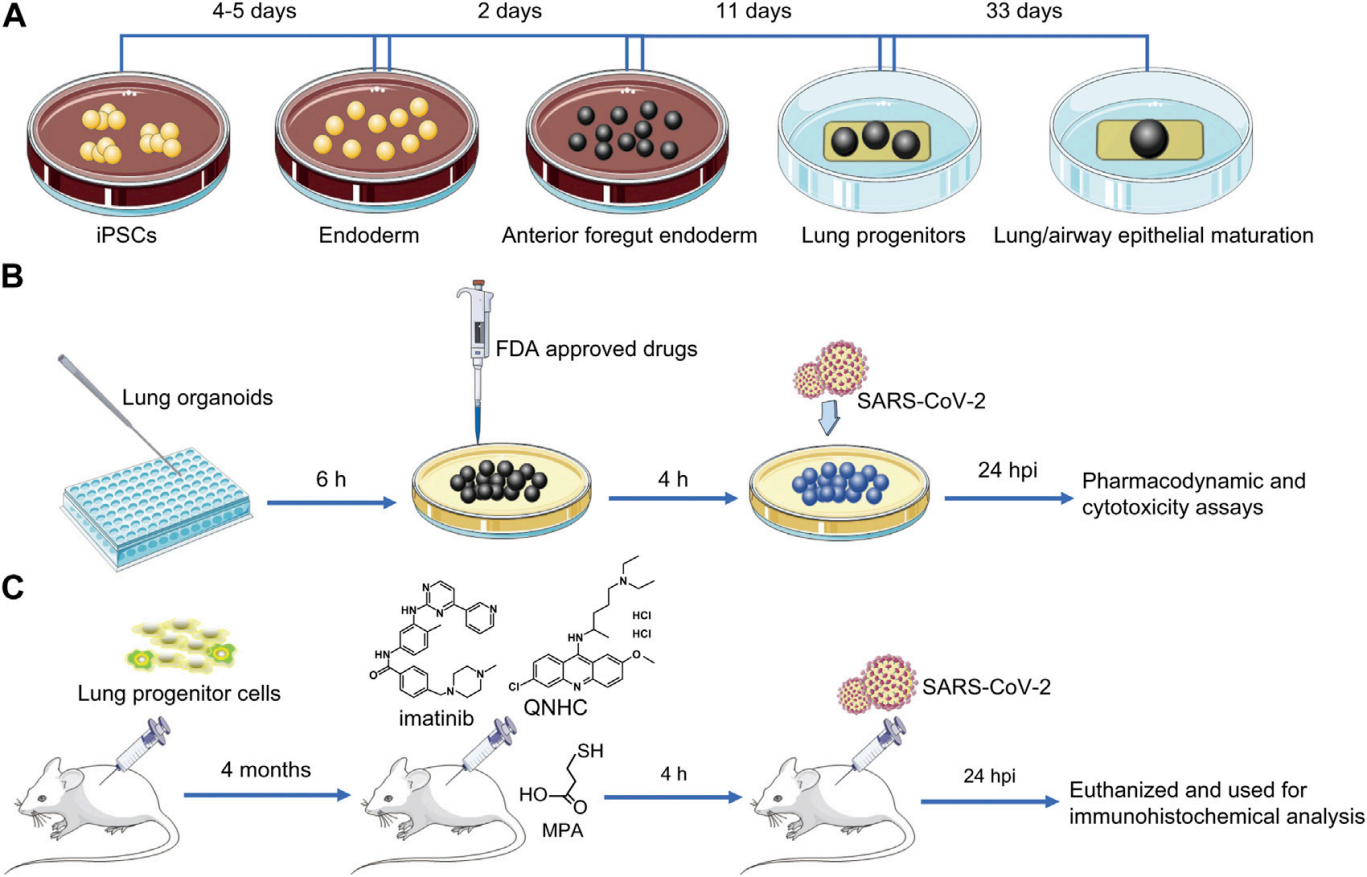
Construction of iPSC-derived lung organoids and their application in COVID-19 drug screening. **(A)** *In vitro* targeted induction of iPSCs into definitive endoderm, then into anterior foregut endoderm, and ultimately into lung organoids using a combination of cytokines and small molecules. **(B)** Transfer iPSC-derived lung organoids into 384-well plates and culture for 6 h to allow clumping. Lung organoids were treated with selected FDA-approved drugs for 4 h, and efficacy and cytotoxicity of drug candidates were analyzed after SARS-CoV-2 pseudovirus infection for 24 h **(C)** Lung progenitor cells were injected subcutaneously into NSG mice to assess the antiviral activity of the drugs *in vivo*. After 4 months of growth and maturation, imatinib, MPA, or QNHC were injected into the xenografts, which were infected with SARS-CoV-2 pseudovirus 4 h later. After 24 h of persistent infection, mice were euthanized and used for immunohistochemical analysis. IPSC, induced pluripotent stem cells; COVID-19, coronavirus disease 2019; SARS-CoV-2, severe acute respiratory syndrome coronavirus 2; FDA, Food and Drug Administration; MPA, mycophenolic acid; QNHC, quinacillin hydrochloride.

Models of lung organoids can be used to study the mechanism of COVID-19 lung injury and screen for therapeutic drugs. Prior to this, drugs were screened by cell lines infected with SARS-CoV-2, but cell lines, in which it is difficult to simulating the pathophysiological behavior of tissues and cells following viral infection, and human organoids infected with SARS-CoV-2 can simulate real organ signals to a certain extent, so the drugs screened by this method are likely to have more clinical significance. In 2020, Han’s group was the first to disclose the use of lung organoid models to explore COVID-19 and test for therapeutic medicines. The lung organoid models provided the necessary conditions for SARS-CoV-2 infection of AT2 cells. Transcriptional analysis after SARS-CoV-2 infection revealed significant chemokine and cytokine production, with almost no type I/III IF signal. In these lung organoid models, upregulation of IL-17 signal pathway was similar to that of lung tissue in patients infected with COVID-19. The findings indicated that human lung organoids can be utilized to investigate SARS-CoV-2 infection and pathogenesis in details. The researchers investigated the effectiveness and cytotoxicity of FDA candidate medications at various doses and discovered that imatinib, mycophenolic acid (MPA), and quinacillin hydrochloride (QNHC) inhibited SARS-CoV-2 infection in a dose-dependent manner rather than cytotoxicity. They injected lung progenitor cells subcutaneously into NSG mice and found that after 4 months of growth and maturation, when imatinib, MPA, and QNHC were injected into the xenografts infected with SARS-CoV-2 pseudovirus, all three drugs were able to prevent SARS-CoV-2 pseudovirus infection of AT2 cells (Han et al., 2020; Han et al., 2021). Construction of iPSC-derived lung organoid and its application in screening drugs for COVID-19 is shown in Figure 4. Most current investigations of SARS-CoV-2 infection in organs do not involve immune cells, and adding immune cells to cultures will help us understand the pathophysiology of SARS-CoV-2 infection in the future, which may be a direction we should consider when modeling COVID-19 related diseases in the future.

## 4 Ethical issues in the research and use of iPSC-based organoid models

The discussion on the ethical aspects of organoid models based on iPSCs usually covers two aspects. On one hand, organoid models have their own unique advantages as favorable alternatives to experimental animal models, thus the ethical issues related with the use of animal models are circumvented. On the other hand, organoid models, especially intracerebral organoids, have their own unique ethical dilemmas due to their ability to reproduce the tissue structure of the human body, which require further discussion of the ethical boundaries and the search for solutions.

The development of organoid models has had a significant influence on the current ethical debate around animal research. Many people believe that animal experimentation should be allowed only if the 3 R’s are strictly followed (replacement, reduction, and improvement) (Franco and Olsson, 2014). Despite the general acceptance of these principles, ethical conflicts still exist in many specific instances (Franco et al., 2018). A good alternative to animal models is therefore needed to resolve these complex ethical dilemmas. Organoid technology is a long-awaited substitute for animal experimentation. Several countries currently have animal experimentation restrictions in their animal research regulations, which implies that researchers must always work to decrease, improve, and replace animal experimentation (Olsson et al., 2007; Fischer et al., 2021). In the future, as there is advancement in organoid model technology with newer and more precise models being developed, the rationale for using animal experiments instead of organoid models may need to be articulated depending on the circumstances. In this context, organoid models are evidently an attractive alternative.

Conversely, organoid models, especially intracerebral organoid models, have their own ethical dilemmas to discuss. The majority of these ethical quandaries are concerned with whether *in vitro* brain organoids would have any awareness that would warrant moral consideration. According to Nita Farahan and others, when *in vitro*-generated brain organoids grow in size and complexity, they may achieve human-like perceptive capacities, such as the ability to feel things, store and retrieve memories, perception of self-awareness, *etc.* (Farahany et al., 2018; Lavazza and Massimini, 2018). Modern brain organoids do not yet have sophisticated neural networks capable of sensory input and motor output, so the likelihood of existing brain organoids having a mind is extremely low (Munsie et al., 2017; Lavazza and Massimini, 2018). If future advancements in culture conditions allow brain organoids to develop mature human brain-like neurological functions, and *in vitro* brain organoids develop a consciousness that necessitates ethical considerations, then research and therapeutic uses of these brain organoids will pose an even bigger ethical problem (Glannon, 2016; Lavazza and Massimini, 2018).

However, these potential ethical difficulties do not imply that all existing brain-like organ research should be prohibited. Further ethical difficulties may arise as the field develops. For example, research on fused neurological organoids, which try to link brain organoids to other neural tissues, may become technically feasible in the near future, posing significant ethical challenges (Birey et al., 2017; Xiang et al., 2017). This raises the ethical dilemma of whether it is appropriate to enable the creation of brain organoids with tactile or visual capabilities. When increasingly complicated brain organoids are created, we may question if additional organoid models with human sensory capabilities may be created and employed, and if so, where are the boundaries of application, and what should be done with these highly similar human tissue organoids at the end of the experiments.

We must also underline that *in vivo* and *in vitro* organoid research based on iPSCs must adhere to established ethical norms and monitoring methods. It has been proposed that these experiments be carried out gradually, and that any changes in the host animal’s physiology and behavior be thoroughly observed (Munsie et al., 2017; Farahany et al., 2018). Experiments related to organoid transplantation, on the other hand, should be monitored and regulated. Currently, all experiments in which human cells of any sort are transplanted into living animals adhere to scientific and ethical guidelines for animal research. Indeed, the International Association for Stem Cell Research has made recommendations on how such research should be carried out (Hyun et al., 2007; Daley et al., 2016; Kimmelman et al., 2016). Although organoid models do not currently raise substantial and unique ethical issues, we need to continue to closely monitor ongoing experiments and developments in the agenda that may require updated and more detailed ethical analysis. In the meantime, we look forward to hearing public discussion of relevant issues so that key public perspectives can be incorporated into the process of refining the ethical issues associated with organoid models, thereby optimally utilizing this technology to address the dilemmas currently encountered in our scientific research.

## 5 Current challenges in organoid modeling based on iPSCs

### 5.1 Sourcing of clinical-grade iPSCs and construction of a universal cell line biobank

For iPSCs that are entirely derived from patients, the burden of demonstrating immune compatibility and donor safety is much lower than with ESCs (Condic and Rao, 2008). Though iPSCs-based regenerative therapy can avoid the long-term use of immunosuppressants in patients, it is still subject to many clinical modalities, which should be considered when used for human treatment. Patients treated with derived-iPSCs are required to be tested for persistent and highly virulent infections (such as COVID-19) in addition to prescribed relevant pathogen testing, and the iPSCs are required to be reproducibly and efficiently generated with a zero genomic “footprint” to avoid the tumorigenic potential of the final product. To avoid the reagents or medium used in the production process affecting the reproducibility of the final product. or causing infection or inflammation when transplanted into patients, the auxiliary materials used must comply with standard manufacturing practices, and appropriate product quality testing, *etc.* (Jha et al., 2021). These challenges will be addressed as more scientific knowledge is gained from preclinical studies and clinical development of regenerative medicine products increase (Azuma and Yamanaka, 2016).

There are other challenges in the application of iPSCs to CRT, in addition to the issues we are relatively familiar with, such as tumorigenicity, heterogeneity of iPSC lineage phenotype cells, cell line variation, cell number requirements, genetic instability and lack of maturation of derived phenotype cells (Doss and Sachinidis, 2019). These challenges are mainly related to selecting appropriate donor cell types for the reprogramming of iPSCs; dermal fibroblasts in skin biopsy tissues, T-cell in peripheral blood, renal tubular cells collected in a urine sample, and keratinocytes separated from extracted hair are the most common sources of iPSCs (Aasen et al., 2008; Haase et al., 2009; Dambrot et al., 2013). Several studies have found that iPSCs maintain some residual epigenetic memory obtained from their donor cells, which may lead to biased differentiation into specific types of cells depending on the source of the donor cells (Ghosh et al., 2010; Polo et al., 2010; Bar-Nur et al., 2011; Kim et al., 2011). According to some reports, the remaining epigenetic memory of cells decreases as they travel through successive generations in culture (Ghosh et al., 2010; Nishino et al., 2011). An important issue in the selection of donor cell types is that certain types of donor cells, such as dermal fibroblasts and blood cells generated from skin biopsies, may come with more mutations and chromosomal abnormalities as a result of exposure to UV light and increased cell turnover (Musunuru et al., 2018; Strassler et al., 2018). This is an issue that needs to be considered in the construction of iPSCs cell biobank. At least the information such as gender, age, source cell type, *etc.*, Should be recorded precisely.

### 5.2 Lack of maturation of iPSC-derived phenotypic cells

One of the major challenges in using patient-specific iPSCs derived from clinically relevant phenotypic cells as an *in vitro* disease model for high-throughput drug discovery, safety pharmacology, or CRT is that the majority of these iPSC-derived phenotypic cells exhibit immature functional characteristics similar to their respective embryonic or fetal phenotypic cells and also contain heterogeneous mixtures of phenotypic subtypes in varying numbers (Chun et al., 2015). Developing consistent and accurate disease models with distinct cell properties and homogenous phenotypic cell populations is a critical precondition for high-throughput therapeutic applications (Doss et al., 2012). Early-onset disease models based on iPSCs have been widely employed and have proven to be useful models for early-onset disorders (Moretti et al., 2010; Bellin et al., 2013). Nevertheless, some of these disease models failed to delay the disease because iPSC-derived related phenotypic cells lacked adult maturation features that expressed disease phenotypes, and these cells exhibited more embryonic or fetal-like traits. There are numerous ways to stimulate the development of these initial iPSC phenotypic cells, including the use of mitochondrial stress inducers, protein degradation inhibitors, and pyrrolidines. Although these techniques generated some aging of iPSC-derived phenotypic cells, the maturity of these cells was modest because aging and maturation appeared to take distinct paths (Studer et al., 2015; Ho et al., 2016). Another strategy was to directly transform somatic cells into therapeutically relevant phenotypic cells in order to maintain cell senescence signals and probable maturation (Qian et al., 2012). But this direct reprogramming *in vitro* did not have the other advantages of organoids.

### 5.3 Limitations of the organoids themselves

As a new research model, organoids have many advantages, but there are still some shortcomings. Although the 3D environment of iPSC-derived organoids is superior in terms of physiological relevance, the relative simplicity of today’s organoid culture conditions compared to *in vivo* environments, such as fewer cell types, *etc.*, we must admit that organoids will not be very physiologically close to the native microenvironment (although it will be closer than 2D cultured cells). Although organoids have promising properties, their widespread use is subject to various limitations that have not been overcome, including lack of high-fidelity cell types, restricted maturation, abnormal physiology and stability. (Andrews and Kriegstein, 2022), these characteristics may limit their reliability in some applications.

The absence of mesenchymal tissue, blood vessels, and immune cells is a significant intrinsic constraint of organoid cultivation, for which exploration of co-culture system based on organoid culture is needed. Workman et al. (Workman et al., 2017) co-cultured small intestinal organs with neural crest cells derived from human PSC, and found the distribution of functional nerve cells in the small intestinal structure, indicating the possibility of a co-culture system. Some investigators co-culture peripheral blood lymphocytes with tumor organs to obtain T-cell with tumor-killing ability, which can be used in immunotherapy (Dijkstra et al., 2018). However, a more complete co-culture system of various tissues or cells needs to be further developed, and so far, almost all tumor organs are of epithelial origin. Whether the current organoid culture method can be used for other tumors of non-epithelial origin remains to be explored, organoids close to the native microenvironment also need to be further developed.

The external deficiency of organoids culture is that, substitutes of extracellular matrices such as matrix glue, basement membrane extract, or fetal bovine serum are used. Although ECM maintains cell viability and has biological activity (Varzideh et al., 2022), it contains some uncertain components that may affect the results of drug experiments. Also, there may be batch-to-batch variation and growth factors (Schutgens et al., 2016). Studies have shown that adding serum is not conducive to the long-term growth of human pancreatic organs (Seino et al., 2018). Rat-derived extracellular matrix substitutes are used to culture small intestinal organs. However, this synthetic matrix needs to be improved to improve production efficiency and be suitable for other organs. Water-soluble WNT antagonists used for the cultivation of various organs have been created (Gjorevski et al., 2016), which can become serum-free substitutes of WNT conditioned medium (Seino et al., 2018). The impact of these extracellular matrix substitutes should be considered and appropriately substituted in organoid-related studies.

## 6 Conclusion and future perspectives

Since the establishment of iPSC technology, it has provided a powerful new method for defining and treating diseases, because iPSCs represent a paradigm shift: it allows for direct observation and treatment of relevant patient cells, reveals new relations of gene expression, and broadens and deepens people’s understanding of the development of various diseases (Musunuru et al., 2018). The emergence and continuous innovation of new technologies represented by 3D organoids, animal chimeras, and others will further promote the development of disease models and therapy using iPSCs (Rowe and Daley, 2019).

The iPSCs-based organoids model can be cultured and passaged for a long time, with a stable genotype, which can be frozen and stably cultured after resuscitation, thus providing the possibility for establishing a biological organ bank. By expanding the biobank of similar organs, we can obtain various types of similar organ models and tumor models (Giandomenico et al., 2019). After genome sequencing and expression profiling, guidance at an individual level, such as disease susceptibility, efficacy prediction, and regenerative medicine can be provided, together with enough data to support large-scale experimental research (Obendorf et al., 2020). Organoid culture efficiency and the construction of a network-based drug screening platform allow for personalized and precise drug screening within the clinical treatment time frame, which can be further applied to translational medicine and individualized treatment (Chen, 2020; Park et al., 2021). With the global spread of the COVID-19 epidemic, more and more people have suffered physical injuries in different aspects and degrees. And the construction of different *in vitro* organoids from patients with COVID-19 can explore the damage mechanism of different organ types, and propose strategies for further development of research programs for formulating treatment regimens, at the same time, screening and evaluation of the efficacy of currently widely used drugs can be done (Kanimozhi et al., 2021; Larijani et al., 2021). Although there are many technical difficulties to be overcome in the treatment of patients with severe COVID-19 using iPSC cell therapy, it has significant clinical implications (Li et al., 2022). We cannot ignore the limitations that have not been overcome, no model system is flawless, and organoids are no exception; they must be used with a clear grasp of their limitations (Andrews and Kriegstein, 2022). Overall, iPSCs technology and organoid technology are constantly being optimized, and organoid is expected to become an ideal carrier for future life science research and further promote the advancement of clinical medicine.

## Figures and Tables

**Table:** 

**iPSCs**	induced pluripotent stem cells
**ESCs**	embryonic stem cells
**PD**	Parkinson’s disease
**AD**	Alzheimer’s disease
**SARS-CoV-2**	severe acute respiratory syndrome coronavirus 2
**COVID-19**	coronavirus disease 2019
**hPSCs**	human pluripotent stem cells
**EB**	embryoid body
**APOE**	Apolipoprotein E
**iPSC-LBs**	iPSC-derived liver buds
**CF**	cystic fibrosis
**F508del**	deletion of phenylalanine at position 508
**CNS**	central nervous system
**CFTR**	CF transmembrane conductance regulator
**FGF**	fibroblast growth factor
**OCA**	obeticholic acid
**BMP4**	bone morphogenetic protein 4
**CS**	cytokine storm
**BRD**	bromodomain-containing protein
**BETi**	bromodomain and extra-terminal family inhibitors
**FDA**	Food and Drug Administration
**ACE2**	angiotensin-converting enzyme 2
**AT2**	alveolar type 2
**SFTPB**	surfactant protein B
**MPA**	mycophenolic acid
**QNHC**	quinacillin hydrochloride
**CRT**	cell replacement therapy
